# Dear reviewers: Responses to common reviewer critiques about infant neuroimaging studies

**DOI:** 10.1016/j.dcn.2021.101055

**Published:** 2021-12-27

**Authors:** Marta Korom, M. Catalina Camacho, Courtney A. Filippi, Roxane Licandro, Lucille A. Moore, Alexander Dufford, Lilla Zöllei, Alice M. Graham, Marisa Spann, Brittany Howell, FIT’NG, Sarah Shultz, Dustin Scheinost

**Affiliations:** a Department of Psychological and Brain Sciences, University of Delaware, Newark, DE, USA; b Division of Biology and Biomedical Sciences (Neurosciences), Washington University School of Medicine, St. Louis, MO, USA; c Emotion and Development Branch, National Institute of Mental Health, Bethesda, MD, USA; d Institute of Visual Computing and Human-Centered Technology, Computer Vision Lab, TU Wien, Vienna, Austria; e Department of Psychiatry, Oregon Health and Science University, Portland, OR, USA; f Department of Radiology & Biomedical Imaging, Yale School of Medicine, New Haven, CT, USA; g A.A. Martinos Center for Biomedical Imaging, Department of Radiology, Massachusetts General Hospital, Boston, MA, USA; h Department of Psychiatry, Columbia University Irving Medical Center, New York, NY, USA; i Fralin Biomedical Research Institute at Virginia Tech Carilion, Department of Human Development and Family Science, Virginia Polytechnic Institute and State University, Roanoke, VA, USA; j Division of Autism & Department of Pediatrics, Emory University School of Medicine, Atlanta, GA, USA; k Marcus Autism Center, Children’s Healthcare of Atlanta, Atlanta, GA, USA; l Department of Biomedical Imaging and Image-guided Therapy, Computational Imaging Research, Medical University of Vienna, Vienna, Austria

**Keywords:** Baby, MRI safety, MRI acquisition, MRI processing, Brain development, FIT’NG

## Abstract

The field of adult neuroimaging relies on well-established principles in research design, imaging sequences, processing pipelines, as well as safety and data collection protocols. The field of infant magnetic resonance imaging, by comparison, is a young field with tremendous scientific potential but continuously evolving standards. The present article aims to initiate a constructive dialog between researchers who grapple with the challenges and inherent limitations of a nascent field and reviewers who evaluate their work. We address 20 questions that researchers commonly receive from research ethics boards, grant, and manuscript reviewers related to infant neuroimaging data collection, safety protocols, study planning, imaging sequences, decisions related to software and hardware, and data processing and sharing, while acknowledging both the accomplishments of the field and areas of much needed future advancements. This article reflects the cumulative knowledge of experts in the FIT’NG community and can act as a resource for both researchers and reviewers alike seeking a deeper understanding of the standards and tradeoffs involved in infant neuroimaging.

## Highlights

• The field of infant MRI is young with evolving standards.
• We address 20 questions that researchers commonly receive reviewers.
• These come from research ethics boards, grant, and manuscript reviewers.
• This article reflects the cumulative knowledge of experts in the FIT’NG community.

## 1 Introduction

Over the past three decades, infant neuroimaging (ages 0–12 months) has gained increasing attention for its groundbreaking insights into early human brain development and the neurodevelopmental origins of health and disease. Infant magnetic resonance imaging (MRI) has emerged as an incredibly valuable tool, with several landmark studies—the developing Human Connectome Project (dHCP) (Hughes et al., 2017), Baby Connectome Project (BCP) (Howell et al., 2019), and HEALthy Brain and Child Development (HBCD) (Morris et al., 2020), among others—launched in recent years that are poised to yield important new discoveries.

Despite these considerable successes, the field of infant neuroimaging is still nascent. Relative to the field of adult neuroimaging, which has well validated tools and widely accepted best practices for data collection and analysis (Nichols et al., 2017), infant neuroimaging is still establishing its field standards and new methodologies optimized for infant populations are continuously being developed and refined. In the absence of widely accepted best practices, even reasonable methodological choices can be subject to critique at every stage of the review process, from the ethics review of research protocols, to grant proposals and manuscripts. This scrutiny is critical to the success of our field as we strive to develop field-wide standards that ensure rigor and reproducibility. And yet, critiques also have the potential to restrict growth, particularly when levied without appreciation for the challenges inherent in making data acquisition and analytic decisions in an emerging research field. How then can reviewers and researchers responsibly address concerns about limitations and tradeoffs inherent to most methodological choices, without unduly restricting opportunities for new discoveries and growth?

This article addresses this question by examining common reviewer critiques in the context of structural and functional MRI (fMRI) infant research—as opposed to clinical—studies. Key citations relevant to each critique are provided in Table 1. These critiques are organized broadly by when they come up during the lifespan of a study, although some critiques are applicable to more than one section. They include questions about (a) infant safety from research ethics reviewers, (b) questions about study planning and acquisition from grant reviewers, and (c) questions that arise after the data are acquired and analyzed from manuscript reviewers. For the sake of brevity, we focus our responses on special considerations for studying at-term, healthy infants. Our goal in reviewing these critiques is not to argue that they are unwarranted (indeed, they highlight major issues plaguing the field), nor is it to offer prescriptive guidelines. Instead, we aim to foster a more productive dialog between investigators and reviewers and have crafted each response to act as a resource for both researchers and reviewers alike. We also seek to ensure methodological transparency and rigor while acknowledging the lack of gold standards and identify reasonable pathways to a more robust and mature field with established standards for collection, processing, and analysis of infant neuroimaging data. Our responses reflect the current literature and authors’ cumulative knowledge and experience.

**Table 1:** Key citations that address common reviewer questions, organized by question number and topic. SAR = specific absorption rate; MRI = magnetic resonance imaging; dHCP = the developing Human Connectome Project; ComBat = combine batches; GAM = generative additive model; EEG = electroencephalography.

Question (s)	Key Citations
Q1-Q7. Imaging procedures, infant comfort, hearing protection, and safety monitoring	• Infant imaging procedures: (Howell et al., 2019, Raschle et al., 2012) • Quiet scanning: (Glans et al., 2021) • SAR in infants: (Malik et al., 2015)
Q8. Long-term risks	• MRI safety in infants: (Tocchio et al., 2015) • Safety of repeated MRI in children: (Holland et al., 2014)
Q9. Incidental findings	• dHCP incidental findings and outcomes: (Carney et al., 2021)
Q10. MRI hardware	• Dedicated neonatal imaging systems: (Hughes et al., 2017, Voelker, 2017)
Q11. Harmonizing across scanners	• ComBat-Linear & ComBat-GAM: (Pomponio et al., 2020) • Traveling subject: (Yamashita et al., 2019)
Q12. T1 versus T2 anatomical imaging	• Review of neonatal MRI: (Dubois et al., 2020)
Q13-Q14. Small sample sizes	• Deep phenotyping collaboratives: (Calkins et al., 2015) • Small sample sizes in neuroscience: (Button et al., 2013)
Q15. Infant imaging for studying brain–behavior associations	• Predicting autism from the infant brain: (Emerson et al., 2017) • Infant fMRI as a model system: (Ellis and Turk-Browne, 2018)
Q16. Infant sleep and fMRI	• Comparison of infant and adult sleep fMRI: (Mitra et al., 2017)
Q17. Neuronal-hemodynamic coupling in infants	• Review of neurovascular coupling development: (Kozberg and Hillman, 2016) • Simultaneous EEG–MRI in infants: (Arichi et al., 2017)
Q18. Measuring myelin in infants	• Reviews: (Dubois et al., 2014; Gilmore et al., 2018; Qiu et al., 2015)
Q19. Data processing	• T1/T2-weighted: (Adamson et al., 2020; Dai et al., 2013; Zöllei et al., 2020) • Diffusion: (Bastiani et al., 2019) • Resting State: (Fitzgibbon et al., 2020)
Q20. Open science practices	• Best practices in data analysis and sharing: (Nichols et al., 2017) • Guide to working with open-source datasets: (Horien et al., 2021)

## 2 Study protocol ethics board reviewers: questions about infant safety

As pilot data are often required for grant proposals, the first point at which infant neuroimagers are typically faced with reviewer comments is when submitting a protocol to their ethics board. Critiques typically focus on issues related to safety, rather than scientific concerns.

Below *(Q1*–*Q9*) is a set of typical ethics board protocol questions and answers about infant safety in the context of research scanning.

Q1 *What monitoring procedures will be put in place to ensure the safety of infant participants during scanning?*

While procedures vary with local and national ethics requirements, two common strategies have been previously used for monitoring an infant during scanning. These strategies may be applied together or separately, depending on the age of the infant, available hardware, and MRI center policies. The first relies on direct physiological monitoring (i.e., electrocardiography, pulse oximetry, thermometry) of the infant, possibly with a clinician present to read these signals, and is most commonly used with infants younger than 6 months. However, this procedure requires MRI-safe external monitors that can fit comfortably on an infant, which may not be available at all MRI facilities. For some scanning centers, a video and/or audio feed of the infant can be broadcast to a variety of locations (e.g., control room, waiting room, etc.), providing infant monitoring without requiring that researchers and parents be in the scanner room itself. The second strategy is to have a researcher remain next to the scanner to provide direct visual monitoring of the infant. This strategy is particularly useful for infants older than 6 months who may attempt to struggle out of a swaddle (see *Q5*) upon waking.

As policies vary by institution, researchers should seek to clarify local policies and available monitoring devices prior to submitting a study protocol or grant proposal, and the guidance here may not wholly satisfy these local requirements.

Q2 *MRI scanners are very loud during image acquisition. How will you ensure the infant’s hearing is protected for the duration of the scan?*

**Fig. 1:**
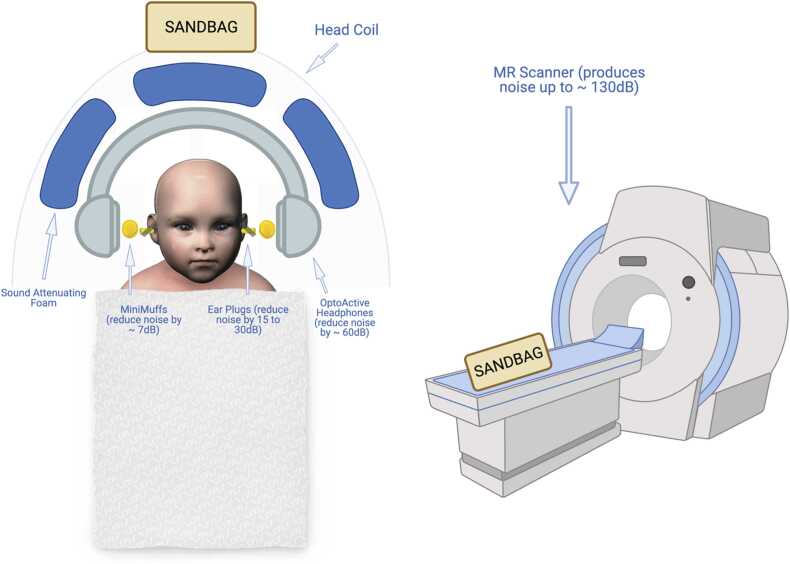
Diagram of infant hearing protection devices (HPDs) commonly used in research MRI scanning. Ear plugs are inserted into the infant’s ear, reducing noise levels by 15–30 dB. On top of the earplugs, sound attenuating foam can further reduce exposure by approximately 7 dB. Finally, passive MR-compatible earmuffs or MR-compatible active noise canceling headphones further dampen sounds by up to 37 and 60 dB, respectively. Sound attenuating foam can be placed between the head and the coil, or on the walls of the MRI tunnel. Sandbags (or padding) can be placed on the scanner bed or around the head coil to secure the infant, hold equipment in place, and reduce scanner table vibration during imaging (see *Q5*).

Current MRI sequences can result in peak noise levels ranging from 122 to 131 decibels (dB) (Foster et al., 2000) with an average noise intensity measured at 110–115 dB across sequences (Radomskij et al., 2002). Hearing loss can begin with 50 min of exposure to sounds at or above 95 dB (CDC, 2019), making dampening these sounds imperative to the safety and comfort of the infant. Although the use of multiple layers of hearing protection does not reduce the noise level by the cumulative number of decibels associated with each noise reduction rating, it is still helpful to use several layers of Hearing Protection Devices (HPDs) for added protection (see Fig. 1). Earplugs (either foam or silicone) inserted into the infant’s ear can reduce noise levels by 15–30 dB (McJury, 2021). In addition to earplugs, sound attenuating foam pads (e.g., Mini-muffs or pads cut from larger sheets of foam) reduce exposure by around 7 dB (Abujarir et al., 2012). Finally, passive MR-compatible earmuffs or MR-compatible active noise-canceling headphones—adapted for an infant’s head— dampen sounds by up to 30–37 dB or 60 dB respectively (Ravicz and Melcher, 2001), depending on the manufacturer. For example, OptoACTIVE technology can dampen sound by more than 40 dB (OptoAcoustics, Israel). In total, these HPDs allow researchers to keep noise exposure below the levels that may induce hearing loss.

One limitation of these commonly used HPDs is that they require proper initial placement on the infant for effective noise reduction and, unfortunately, are not equipped with a mechanism to gauge if proper placement is achieved and maintained throughout a scan session. This is of particular concern when working with infant participants who cannot provide feedback as to whether an effective headphone, earplug, or earmuff seal has been achieved or whether placement has been compromised (due to, for example, participant motion which may displace HPDs and cause discomfort). To mitigate this concern, a sound attenuating acoustic hood can be inserted into the scanner bore that can maintain 16–22 dB of sound attenuation in the event of HPD displacement (Nordell et al., 2009). Recently, research groups have also begun using MRI-compatible microphones to measure in-ear sound levels during MRI scans. This allows researchers to detect and address HPD displacement within seconds, thereby limiting infant exposure to elevated sound levels (Valente et al., 2015). It is notable, however, that such systems are often expensive and therefore are not widely adopted yet. Another strategy is to design quieter sequences by limiting the performance of magnetic field gradients during image acquisition (e.g., Whisper mode, Quiet Suite) (Glans et al., 2021). While this approach successfully reduces noise intensity, it can reduce scan quality and lengthen acquisition time.

Q3 *Are there additional concerns regarding specific absorption rate (SAR) limits for* infants*?*

MRI scanners have safeguards to ensure that the SAR—the rate at which the body absorbs radiofrequency energy during MR scanning—stays within a healthy range according to the individual’s height, weight, and age. For most manufacturer-provided sequences, the scanner will not allow the sequence to begin if the scanner predicts that the SAR will exceed established limits. Recent work suggests that neonates may experience 25–50% of the SAR that adults experience from the same sequence (Malik et al., 2015) suggesting that the conservative limits placed by default are even more conservative for infants.

Accurate height (or length in the case of infants), weight, and age information is needed to properly calculate SAR limits. Thus, collecting height/length and weight before a scan is recommended as opposed to relying on parent report. Some facilities have strict policies on protected health information which prohibits entering the infant’s exact birthdate into the scanner. This policy is immaterial for imaging older participants for whom a month or months are not meaningful, however infants can change noticeably within a matter of weeks. In this case, semi-randomized birthdates as close to the exact birthdate as possible (e.g., entering Dec 1 rather than Dec 3 or Dec 5) or birthdates rounded to the Sunday of the infant’s birth week may be used. If accurate participant measurements are provided, there is little concern regarding SAR limits for infants of any age.

Q4 *How will temperature be maintained* at *a comfortable level for the infant?*

In most cases, the clothing layers needed to scan infants safely (see *Q5*) keep them sufficiently warm, and when combined with the heating during scanning can make the infant sweat. Thus, infants can be dressed lightly (i.e., a breathable cotton outfit or just a diaper) with a wearable blanket or swaddle that can either be easily removed or unfastened if the infant becomes too warm. A blanket or swaddle that unfastens from the bottom is particularly helpful for accessing the infant’s feet if using a pulse oximeter or other physiological monitoring system. During scanning, infant temperature can be monitored by a researcher in the scanner room who is watching the infant for signs of sweating or shivering, or by using an optical thermometer, readable from the MRI control room. If the infant appears too hot or cold, adjustments can be made in real time by the researcher in the scanner room, or the protocol can be stopped as needed (see *Q6*).

Q5 *How will* the *infant be kept from rolling off the scanner table?*

**Fig. 2:**
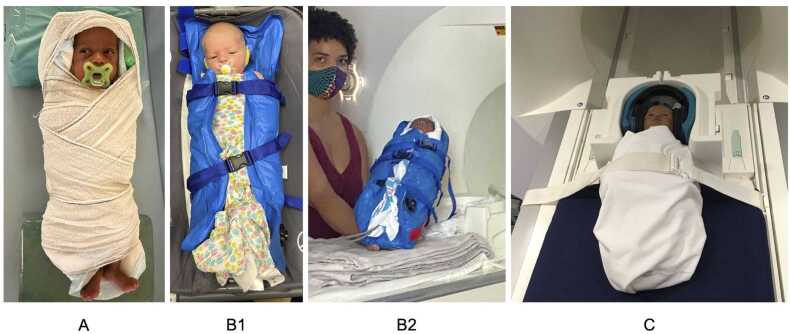
Immobilization approaches common for infant scanning. A. Swaddling the infant in an MRI-safe wrap or blanket; B. A vacuum immobilizer on an infant up close (B1) and on the scanner bed with leads attached for external monitoring (B2). C. A strap that prevents awake or older infants from rolling off the table (swaddle is optional).

There are devices, such as immobilizers, that can be used to secure the infant to the table (see Fig. 1, Fig. 2). Once the infant is securely swaddled, the infant can be secured to the scanner bed with padding or sandbags arranged into a cradle or with a strap/buckle that goes across the infant. Although there are significant individual differences, swaddling is the most useful for infants younger than 6 months old, whereas a strap that secures the infant on the scanning table might work better for older infants. For awake infant scanning, time with the infant and parents in the scanner room may be beneficial to get the infant used to the MRI padding and safety straps necessary to prevent the infant from rolling off the bed. With these secure methods and continuous monitoring, there is little concern about the infant rolling over and falling off the table.

Q6 *How will researchers determine when to stop a scan?*

We have found that the decision of when to stop a scan varies based on study goals and the specific needs of the studied population. There are several sources of information for determining when to stop a scan, including measurements from infant monitoring procedures (see *Q1*), infant behavior, and/or feedback from caregivers, technicians, or medical staff. A scan may be stopped or paused if the infant begins to cry, with soft rules about other distress/waking signs such as vocalizing or moving that are tailored to the specific infant. In some imaging facilities, strict limits on scan time exist while others are flexible on time, especially after usual business hours, which can affect how many times a research team will attempt to scan each infant.

Thus, for researchers seeking to determine what policy to establish in their own study, the decision to stop a scan session can be made with input from the family in conjunction with a general protocol to ensure staff hours are protected (e.g., having a specific time in the evening as a hard stopping point), and any special considerations for the specific population being studied.

Q7 *Will caregivers be allowed to remain with the infant during scanning?*

As with *Q1*, procedures will vary by institutes, ethics boards, and imaging facilities. At facilities that permit individuals to stay in the scanner room during operation, caregivers may remain in the scanner room with their infant after being screened for MRI safety and supplied with appropriate hearing protection. If caregivers are not permitted in the scanner room, a video and/or audio feed of their infant may be available to caregivers instead (see *Q1*).

Q8 *Are there any long-term risks associated with infant scanning, particularly when multiple scans across development are planned?*

There is no increased physical risk of MRI in infants as compared to adults (Tocchio et al., 2015), of stillbirth, neonatal death, congenital anomaly, or hearing/vision loss for infants scanned as fetuses (Ray et al., 2016), nor for lower neurocognitive abilities or differences in BMI after collecting multiple MRIs across development in children (Holland et al., 2014). The Food and Drug Administration guidance states that there is no risk for infants scanned using less than 4 Tesla (T) MRI (Food and Drug Administration, 2014). Finally, recent work has shown safe levels of SAR while scanning infants at higher field strengths such as 7 T (Annink et al., 2020).

Q9 *How will incidental findings be handled?*

We have found that many institutions have existing protocols for the management of incidental findings. However, it is important to consider whether existing protocols—often developed for MRIs of adults—are appropriate for infant participants. Researchers must balance the ethical imperative to share clinically meaningful findings while avoiding misinterpretation of benign findings that may cause undue alarm to parents or may encourage them to pursue expensive medical follow up (in countries without nationalized healthcare). One approach is to have infant MRIs read by a radiologist to determine if an incidental finding should be reported to the parents or followed up on clinically. Some institutions may require that all MRI scans be read by a radiologist, while others rely on investigators to identify incidental findings that are then further examined by radiologists. In cases where clinical follow-up is recommended, researchers may seek necessary approvals (from their institution and from the parents) to send the radiologist report to the child’s doctor as needed or appropriate.

Because the prevalence and clinical significance of incidental findings may vary with age, researchers should take care to work with radiologists who have expertise with the specific age range under investigation. While studies of older individuals report prevalence rates of < 4% (Bos et al., 2016, Maher and Piatt, 2015; Morris et al., 2009), the dHCP (*N* = 500) reported a prevalence rate of 47% in asymptomatic term neonates (Carney et al., 2021). Many of these findings are associated with vaginal birth, not considered clinically significant (Carney et al., 2021, Kumpulainen et al., 2020), and not associated with cognitive or behavioral functioning at 18 months (Carney et al., 2021). More work is needed to determine how common incidental findings are at different ages across infancy as well as their associations with long-term outcomes to aid radiologists in determining the seriousness of a given finding in an otherwise healthy child.

Protocols for the management of incidental findings should be clearly communicated to parents throughout their involvement in the study, and especially during the consenting process. During the consent process with caregivers, it is important to emphasize that research MRIs are not collected for the purpose of yielding clinical measures or rendering a medical diagnosis. It is also helpful to prepare the family for the possibility of an incidental finding by telling them that incidental findings can sometimes happen and are often benign.

## 3 Grant reviewers: questions about study planning and data acquisition

Reviewer critiques for grant proposals are often the most comprehensive and include topics such as infant safety, study design, scientific premise, analytical methods, and interpretation of results. As critiques around infant safety were answered above in Section 2 and critiques around methods and interpretations will be answered in Section 4, Section 3 *(Q10*–*Q15*) will focus on questions relating to study design and scientific premise.

Q10 *Can you use hardware designed for adults for infant data collection?*

While MRI hardware designed for adults is rarely optimized for scanning infants, high quality infant neuroimaging data can be acquired with adult hardware with some considerations. The size of a baby's head is much smaller than an adult's, which leads to a lower load of an antenna designed for adults, and thus to lower signal-to-noise ratios. Furthermore, infants have short necks and small heads compared to adults. Many 64-channel head/neck coils designed for adults make it difficult for the infant’s head to be geometrically centered within the coil, potentially leading to decreased contrast between tissue types. While the dimensions of a 32-channel head coil enable researchers to situate most infants more easily in its geometrical center where the signal-to-noise ratio (SNR) is the highest, it yields inferior SNR compared to a 64-channel coil (Keil et al., 2013). Though specialized hardware for infant scanning exists (Hughes et al., 2017), it is rare, expensive, and has its own limitations, including difficulties with positioning larger infants in the small head coil space with full body immobilizers and securing appropriate acoustic protection (see Q2).

Given these considerations, a mix of hardware is found throughout the infant neuroimaging literature. For instance, the BCP used a 32-channel head coil (Howell et al., 2019), the HBCD plans to use a variety of available head coils at each site, and the dHCP uses 32-channel coils that are custom made for infant scanning (Hughes et al., 2017). In addition to head coils, other infant-specific equipment (e.g., pulse oximetry, dedicated ECG electrodes, temperature probe) may be needed. Grant writers should clearly describe the hardware that will be used in their proposed study, citing evidence for its use in the literature.

Q11 *How will you harmonize across hardware changes and/or multiple scanners?*

Consensus holds that hardware should be kept consistent throughout a study, especially in longitudinal studies spanning periods of rapid development (Turesky et al., 2021), such as infancy. However, researchers often cannot control the schedule of hardware changes and must account for these common confounds. Multi-site projects face a similar problem of introducing measurement bias by pooling data across several scanner types and hardware (Cannon et al., 2014, Focke et al., 2011, Panman et al., 2019; Smith and Nichols, 2018; Yamashita et al., 2019). Although efforts to harmonize data across different hardware exist, most of these have focused on adult datasets (Mirzaalian et al., 2018, Pardoe et al., 2016, Yamashita et al., 2019). Given the rapid volumetric growth seen during infancy, coupled with the difficulty of infant MRI data collection, it typically is not feasible to undertake similar harmonization efforts in infants. For example, the traveling-subject design, where the same individual is scanned on all hardware and/or sites, would require scanning infants during the same developmental period (essentially, the same day) to mimic the adult procedure. Infant MRI phantoms or using a traveling adult can help identify differences across different hardware and sites, however these procedures do not guarantee removal of site effects nor have they been compared to a traveling infant study. Although harmonizing acquisition protocols is recommended, it is unlikely that this will completely harmonize data across hardware. Thus, strategies to minimize multisite confounds after data collection may be the most practical. For example, ComBat can be used to decrease scan-related heterogeneity while increasing statistical power and reproducibility (Fortin et al., 2018, Johnson et al., 2007, Pomponio et al., 2020, Radua et al., 2020, Yu et al., 2018). When different groups of subjects are compared, each site should include subjects from both groups. Additionally, adding a “site” covariate in all analyses is another approach to mitigate site effects.

Q12 *Why was the specific anatomical sequence (*i.e*., T1- vs T2-weighted) chosen?*

**Fig. 3:**
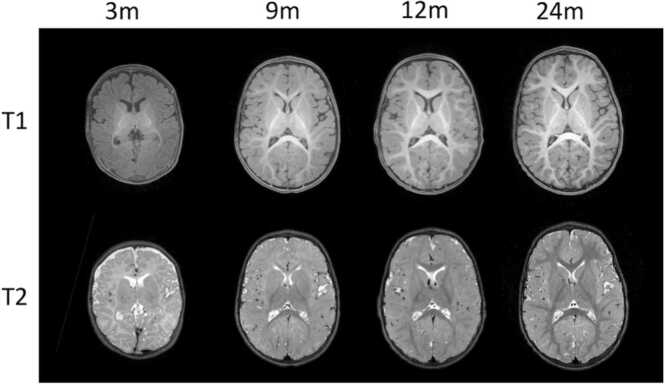
T1- and T2-weighted image contrast from the same individual across the first two postnatal years (scanned at 3, 9, and 12months).

The choice of T1- vs T2-weighted anatomical sequences in infant imaging is not trivial as the signal intensity of and contrast between the gray and white matter change throughout infancy due to white matter development (Dubois et al., 2014, Parazzini et al., 2005). Between 0 and 3 months of age, a reversal of the normal adult contrasts (i.e., gray matter is gray and white matter is white) is observed in T1-weighted MRI, and adult-like contrast is observed more clearly in T2-weighted images compared to T1. Between 4 and 12 months of age, due to non-uniform increasing myelination, poor contrast between gray and white matter is observed non-uniformly across the brain on both T1- and T2-weighted images. The central transition period between newborn and adult-like tissue contrasts (typically occurring between 6 and 10 months of age), is referred to as the “isointense” period in infant imaging (Dietrich et al., 1988), and these data are often the most difficult to process (Zuo et al., 2012). From 1 year and on, adult-like patterns of contrast between gray and white matter are observed in T1-weighted images (Dubois et al., 2020). Fig. 3 shows examples of these three phases of tissue contrast in T1- and T2-weighted images for the same infant.

In the simplest terms, for younger infants (0–3 months), while T1-weighted images can help to identify the early myelination of a few regions (e.g., posterior limb of the internal capsule), T2-weighted images are generally preferred for the delineation of gray and white matter structures, whereas, in older infants (12 months and older), T1-weighted images are preferred (Dubois et al., 2020) since they match the standard for adult imaging. Using a different but optimal sequence at each developmental period could lead to confounds when combining data across these periods, however. Thus, it has been recommended that both T1- and T2-weighted images be acquired to maximize the analytical potential of the data (Howell et al., 2019, Makropoulos et al., 2018). The sequence parameters should also be optimized for infants (for instance with longer inversion times for T1w and echo times for T2w) to deal with the brain tissue immaturity leading to longer T1 and T2 characteristics (Cusack et al., 2018). Various approaches have been developed to use both images in processing (Gui et al., 2012, Prastawa et al., 2005, Wang et al., 2015, Weisenfeld and Warfield, 2009), and recent advances have also shown successful processing of T1-weighted images in infants (Zöllei et al., 2020). Ultimately, while acquiring and processing a T1-weighted image (e.g., a MPRAGE sequence) is ubiquitous in imaging for older children, adolescents, and adults, the anatomical sequences used in infant imaging rely on many factors and the importance of these factors may change based on the interests of the experiment. Another question is whether the spatial resolution should be adapted according to age, to aim for a similar “apparent” resolution of brain structures across ages, according to brain growth. Thus, no single “out of the box” solution for which sequences to acquire exists.

Q13 *Why is the sample size so small?*

Infant MRI studies are expensive and resource-heavy which often leads to smaller sample sizes. For many research aims, collecting data from narrow age bins (e.g., 3- to 4-month-olds) is necessary given the rapid brain (and behavior) changes in early infancy (Knickmeyer et al., 2008). As infants are only eligible for a study for a short time, participant recruitment often starts well before infants reach the age under investigation and often before birth. To quantify the rapid changes in infancy, longitudinal studies often use dense sampling (e.g., scanning every 1–3 months) and need to juggle several age bins concurrently. Furthermore, to keep retention high, additional efforts outside of research visits are required (e.g., small gifts, phone calls)—further taxing resources (Turesky et al., 2021). Finally, collecting data in infants is more difficult than older participants. Parent perceptions about MRI safety in infants and their knowledge and comfort with MRI varies and influences their willingness to participate in infant MRI research (Kohlasch et al., 2021). Getting an infant to sleep in a novel and noisy environment is challenging (Almli et al., 2007, Dean et al., 2014, Nordahl et al., 2007) and often requires reserving the scanner for additional time to account for the infant waking during the scan. These procedures in turn require more staff time than imaging procedures for older populations. Further, a sleeping infant does not guarantee usable data for further analyses (Anderson et al., 2001, Cusack et al., 2018). Some studies have reported the success rate of their scanning procedure in infants (e.g., Dubois et al., 2006). While small sample sizes can still contribute meaningful findings (see *Q14*), these challenges underscore the need for innovations in data collection and sharing across infant neuroimagers to overcome the statistical limitations inherent to small sample sizes.

Q14 *What do small samples contribute?*

Recent consortia and collaborations are generating large repositories of infant data—e.g., BCP, dHCP, IBIS, HBCD—however small studies (i.e., single site studies) are still carried out regularly and are necessary for advancing specific scientific questions. Indeed, the struggle to collect larger datasets is common to imagers across all ages—the median sample size for adult MRI publications remains relatively low (N = 23–24) across the most highly cited papers from 2017 to 2018 (Szucs and Ioannidis, 2020). While large sample sizes are important for addressing research questions such as establishing normative trajectories of brain development (Bethlehem et al., 2021) or investigating associations with small effect sizes (Button et al., 2013, Marek et al., 2020), small studies can play an important and often complementary role in driving discoveries.

Considering the costs and efforts required to obtain infant MRI data, small studies can provide pilot data to generate hypotheses, test proof-of-concept high-risk study designs (Roche-Labarbe et al., 2007, Vanhatalo et al., 2014), or optimize data collection procedures before making a large investment. To this end, it is helpful to report effect sizes and confidence intervals in addition to p-values when applicable. Second, there is growing evidence that ‘deep phenotyping’ and dense sampling will be key to uncovering brain–behavior associations (Adibpour et al., 2017, Calkins et al., 2015, Newbold and Dosenbach, 2021, Robinson, 2012), especially during periods of rapid development. For example, a study utilizing relatively small but dense sampling (i.e., scanning infants every month) and detailed behavioral assessments may capture more meaningful individual differences in developmental trajectories compared to a study utilizing a larger sample of infants with less frequent observations and coarser behavioral measures. Third, small samples can be combined using increasingly accessible techniques such as creating a synthetic cohort (Luby et al., 2019) and quantifying and removing site effects from data analyses (see *Q11*). While challenges exist (see *Q20*), neuroimaging has a rich history of pooling data in this manner (Di Martino et al., 2013, Risacher et al., 2009). Finally, small samples still provide important insight to specific neuroscientific processes that are common across individuals (i.e., studies of inter-individual similarity rather than individual differences) such as visual field or motor mapping (Deen et al., 2017).

Q15 *What can infant neuroimaging tell us about later behavior?*

Ample research suggests that the foundations of behavior in toddlerhood and beyond are present in neonate/early infant brain characteristics (Chen et al., 2021, Dickinson et al., 2021, Girault et al., 2019, Girault et al., 2019; Liu et al., 2021; Overfeld et al., 2020; Saha et al., 2020; Salzwedel et al., 2019; Short et al., 2019; Sket et al., 2019; Thomas et al., 2019; Zuk et al., 2021). Perhaps most importantly, infant neuroimaging data may also be able to predict neurodevelopmental disorders (such as autism spectrum disorder or dyslexia) years before reliable diagnoses can be made (Emerson et al., 2017, Hazlett et al., 2012, Hazlett et al., 2017, Langer et al., 2015).

Additionally, infant neuroimaging can aid in our understanding of the brain and behavior by providing a model system for understanding how the brain works (Dehaene-Lambertz and Spelke, 2015). For example, as most experiences are new for an infant, studies of perceptual learning—the improvement in discrimination abilities due to experience—conducted in infants do not need to account for the extensive learning throughout a participant’s lifetime and, thus, may have greater power to test proposed theoretical explanations of learning (Ellis and Turk-Browne, 2018).

## 4 Manuscript reviewers: questions that come up after data are acquired and analyzed

Through the life of a study, the final place infant neuroimagers receive comments and critiques is from journal editors and reviewers. Like grant reviewers, all aspects of the study are open for comment. However, most focus on the methodological choices used in analyzing the data and the interpretation of the results in the context of the larger literature. Common critiques on these topics are presented below *(Q16*–*Q20*).

Q16 *How does sleep impact infant functional data?*

In contrast to older participants, most infants are primarily scanned during natural sleep (Copeland et al., 2021, Dean et al., 2013, Mathur et al., 2007) to minimize motion. Sleep changes the brain’s response to stimuli in both adults and infants (Dehaene-Lambertz, 2002, Larson-Prior et al., 2009, Tagliazucchi et al., 2012). Furthermore, functional connectivity patterns in asleep 6 and 12 months old infants more closely resemble functional connectivity patterns in asleep adults than awake adults (Mitra et al., 2017), suggesting sleep-related effects in infant functional neuroimaging. Further complicating matters, neonates have different patterns of sleep stages compared to adults or even older infants, entering rapid-eye movement (REM) sleep earlier in sleep and staying in REM sleep longer. By three months of age, infants enter non-REM initially and spend less time in REM sleep than neonates (Middlemiss, 2007). Finally, infants of any age may wake during a scan without moving. Together, this suggests that multiple sleep states—rather than a single ubiquitous one—may need to be accounted for in infant scans.

Several approaches for measuring the sleep stage during a scan exist. Behavioral indicators of sleep-wake states can be monitored with MRI-compatible cameras (see *Q1*). Peripheral measures of autonomic nervous system regulation have been used in adults to identify sleep stages, as well (Bunde et al., 2000, Faust et al., 2019, Herzig et al., 2018). Studies using electroencephalography (EEG) to explore the functional network organization of the infant brain have found substantial differences between REM vs. quiet sleep stages (Tokariev et al., 2019). Simultaneous EEG and fMRI are emerging in adults (Horovitz et al., 2008), yet only one study exists in infants (Arichi et al., 2017), presumably due to the added complexity of EEG setup in addition to the already challenging scanning protocol (*Q13*). Additionally, studies have shown the feasibility of scanning awake infants (Biagi et al., 2015, Deen et al., 2017, Ellis et al., 2020, Meek et al., 1998). While none of these approaches are commonplace and sleep-related effects may be present, functional neuroimaging data in sleeping infants can still reliably characterize short and longer-term brain development, with ample research demonstrating that infant fMRI data can predict behavioral outcomes (see *Q15*). Reporting the cognitive state of the infants (e.g., awake or asleep) and information on waking during scanning (e.g., how many infants woke during functional data collection and how many times) may aid in interpretation of results in the context of broader literature.

Q17 *Is neuronal-hemodynamic coupling in infants comparable to adults?*

Considering that fMRI measures local neuronal firing indirectly via shifts in local blood oxygenation, it is important to consider the developmental course and implications of neuronal-hemodynamic coupling when designing and interpreting infant studies. Recent rodent work indicates that adult-like neuronal-hemodynamic coupling is not established in rats until after postnatal day 23 (Colonnese et al., 2008; Kozberg and Hillman, 2016; Kozberg et al., 2013), when rat neurovasculature becomes adult-like in branching and density (Harb et al., 2013). Post-mortem studies of human neurovascular development show that infant humans do not reach adult-like levels of capillary density until after five months of age, and development until that point is nonuniform across cortical layers (Norman and O'kusky, 1986). Further, there is a small but growing body of literature indicating that cerebral blood flow increases across the first year of infancy (Bouyssi-Kobar et al., 2018, Camacho et al., 2019; Liu et al., 2019). Finally, how local blood flow responds to stimuli—e.g., the hemodynamic response function (HRF)—likely changes across the last weeks of pregnancy, the perinatal period and infancy (Arichi et al., 2012) and the age at which the adult-like hemodynamic response emerges remains unknown. Indeed, human task-based fMRI and functional near-infrared spectroscopy studies have identified a mixture of positive, negative, and delayed hemodynamic responses to task stimuli in infants and children under 5 years of age (Arichi et al., 2012, Arichi et al., 2010, Born et al., 2000, Deen et al., 2017, Issard and Gervain, 2018, Meek et al., 1998, Minagawa-Kawai et al., 2011, Yamada et al., 1997). Furthermore, neuronal-hemodynamic coupling and the HRF response may differ not only with age, but also across functional systems (Issard and Gervain, 2018). All of which may affect the power of different paradigm designs (Cusack et al., 2015). Despite these considerations, emerging work using simultaneous EEG-fMRI suggests a tight coupling between electrical activity and hemodynamic response as early as 40 weeks gestation (Arichi et al., 2017). More research is needed to characterize the extent to which neurovascular changes affect the HRF in infancy and to develop methods for estimating the HRF response that are tailored to the specific population and functional system of interest (Baxter et al., 2019).

Q18 *Can you measure myelination in infants?*

As described in *Q12*, young infants have low white matter myelin levels as compared to adults. Nonetheless, researchers can successfully measure white matter tracts and myelin content in infants using a variety of methods including diffusion imaging (Aeby et al., 2013, Brenner et al., 2021, Dubois et al., 2008, Dubois et al., 2006, Humphreys et al., 2020, Hüppi et al., 1998, Lean et al., 2019, Neil et al., 1998, Rogers et al., 2016, Smyser et al., 2016), quantitative T1/T2 mapping (Deoni et al., 2015, Deoni et al., 2012, Melbourne et al., 2016), and myelin water fraction imaging (Dai et al., 2019; Dean et al., 2014; Deoni et al., 2015, 2012, 2011; Melbourne et al., 2016) among others (Carmody et al., 2004, Soun et al., 2017, Weber et al., 2020, Zhang et al., 2019). Sequences created for measuring white matter in adults can be applied to infants with some alterations to acquisition and processing.

While these methods can be readily used to measure myelin in infants, there are several key differences in the collection and analysis of these data in infants relative to adults that should be considered. For example, when collecting diffusion-weighted imaging (DWI), infant researchers often use lower weightings—the degree to which water is measured as opposed to other tissue content for a given volume of data—to improve diffusion estimates in low-myelin/high-water regions (Conturo et al., 1995, Neil and Smyser, 2021), collect more weighted volumes than typically needed for analysis to ensure that enough low-motion data are collected, and intersperse unweighted and weighted volumes across collection to improve rigid realignment since the signal is far more robust in unweighted volumes for young infants. When using sequences that involve collecting multiple image slices at once such as simultaneous-multi-slice (SMS, also referred to as multiband imaging), researchers may also need to extend the pre-scanning time to allow for the infant to startle and settle before the sequence calibrates. Best practices for analytical steps for older populations are also applicable to infant data. For example, infants often move during diffusion sequences, necessitating strict motion control of the data by removing motion-contaminated volumes and applying rigorous motion correction. A common approach for analyzing DWI is to apply a tensor model to each voxel in order to estimate the direction(s) of the myelinated fibers passing through. The typical fiber tracking parameters used to capture white matter tracts in adults may not be able to capture the same fibers in newborn infants. This is due in part to the smaller physical size of the tracts and related partial voluming issues, as well as to variability in the extent of myelination and underlying axonal microstructure along the tracts during this time of dramatic changes in brain development. Further, differences in both the amount of myelin restricting motion in a given voxel (affecting minimum thresholds for tract inclusion) as well as differences in anatomical expectations (e.g., minimum fiber lengths may be too high for the smaller infant brain and maximum lengths may be too long) will affect the parameters used in fiber tracking software. In sum, measuring myelin in infants is possible and there is a rich literature with a variety of methods and tools to enable this research.

Q19 *Why deviate from existing data processing standards designed for adults?*

Standard processing tools designed for adults are not typically optimized for infant data. In children, adolescents, and adults, neuroimaging processing and analysis have historically been performed using SPM (Ashburner, 2012), FreeSurfer (Fischl, 2012), ANTs (Tustison et al., 2014), FSL (Smith et al., 2004), AFNI (Cox, 1996), or some combination of these packages using an integrated framework combined with custom tools (Cieslak et al., 2021, Esteban et al., 2018, Glasser et al., 2013, Hagler et al., 2019). Unfortunately, these tools and pipelines do not work out of the box for infant neuroimaging. Algorithmic parameters and assumptions—tissue priors (Altaye et al., 2008), hemodynamic response functions (Anderson et al., 2001), brain size (Knickmeyer et al., 2008), contrast differences (Makropoulos et al., 2018), different motion artifacts (Baxter et al., 2019)—are not tuned for the anatomy and physiology of an infant (which are also rapidly changing) (Dubois et al., 2020). Despite recent advancements in infant brain processing software development, such as Infant Freesurfer (Zöllei et al., 2020), iBEAT (Dai et al., 2013), M-CRIB (Adamson et al., 2020) or AutoSeg (Wang et al., 2014) for structural data analysis, neonatal diffusion MRI (Bastiani et al., 2019) for diffusion tensor imaging data, and resting-state data processing pipelines (Fitzgibbon et al., 2020), most evaluations of best practices are not explicitly tested on infant data, leaving researchers to extrapolate these results to their young population of interest. Overall, this has led to a disparate array of standards and tools—some being modifications of adult software for infant studies and others being specifically designed for infants—used in infant neuroimaging. In our experience, researchers often rely on what works for them in their specific sample of infants.

While standards will come, researchers currently need to grapple with the lack of standards and the confusion this causes to those both within and outside of the field. First, the well-known robust software packages in the adult neuroimaging literature are inundated with various processing choices, creating a wide range of divergent results (Botvinik-Nezer et al., 2020). This adds yet another layer of difficulty to relating these differences to infant neuroimaging data. For example, a negligible difference in registering adult data using two different algorithms could be much larger (or simply different) in infants. Second, in-house pipelines are rarely tested on independent datasets of different samples and/or ages, using different scanners, and/or different sequences. Consequently, these pipelines are not guaranteed to work outside of the sample they were developed for. Third, aspects of analysis that are taken for granted in other populations (such as a common stereotactic space) do not exist for infant neuroimaging, which is a barrier to comparisons across studies such as via meta-analysis. In addition, a single common space or even a set of spaces may not be sufficient, given the rapid growth over the 1st year of life (Oishi et al., 2019). Lastly, recent studies functional connectivity in infancy have shown low edge-level test–retest reliability (intraclass correlation coefficients, ICCs, below 0.18) for both intra-session (Wang et al., 2021) and inter-session (Dufford et al., 2021) scans. Moderate to good reliability has been shown for whole-brain functional connectivity metrics and certain ICA-derived networks (Wang et al., 2021). DTI-derived measures for neonates were found to have higher test–retest reliability in a study of neonates (ICCs greater than 0.80) (Merisaari et al., 2019). While several structural MRI pipelines for infant data are currently available, there has yet to be a systematic study of test–retest reliability of structural indices including gray matter volume, cortical thickness, or surface area. Nevertheless, through open-science and community building (see *Q20*), these best-practices and standards will be formalized.

Q20 *Are the data and code publicly available?*

The incorporation of open science practices into single-site and smaller studies, including data and code sharing, has been slow but is needed for the field of infant neuroimaging to mature (Gilmore et al., 2020) and for building larger sample sizes (see *Q14*). While infant neuroimaging datasets are publicly-available on repositories like openneuro.org and infant-specific templates are more widely available (e.g., Oishi et al., 2019), several challenges must be addressed for successful integration of open science practices (Poldrack et al., 2017) into infant neuroimaging research.

One barrier to data sharing is a lack of standard file formatting and organization, for which the Brain Imaging Data Structure, or BIDS, is a growing standard (Gorgolewski et al., 2016). Another is the fear of getting “scooped”, especially when the data are challenging to collect (see *Q13*). Data papers (e.g., Jones et al., 2018), which thoroughly describe but do not interpret a dataset, offer a promising solution. A rich assortment of open infant neuroimaging data, primarily from studies of autism, exists on the National Institute of Mental Health Data Archive (NDA). While there have been recent efforts to combine large datasets across the lifespan (Pomponio et al., 2020), they have not typically included infant data, likely due to the unique challenges inherent in harmonizing (see *Q11*) and processing (see *Q19*) these data.

Similarly, code- and resource sharing are important as best practices are primarily spread through word of mouth rather than broader dissemination. There is a robust image analysis community focusing on methods designed for perinatal and infant data (e.g., see https://pippiworkshop.github.io/). Bridging those who create new algorithms and those who collect infant data to share resources (e.g., open-source data for algorithm development and validation; open-source methods to analyze data) will accelerate the creation and use of new tools and, therefore, advance the field. Even with algorithmic improvement, true software packages are needed (Gewaltig and Cannon, 2014). Though some are emerging (e.g., iBEAT (Dai et al., 2013), dHCP pipelines (Bastiani et al., 2019, Fitzgibbon et al., 2020), M-CRIB (Adamson et al., 2020), Infant FreeSurfer (Zöllei et al., 2020), NiBabies (https://github.com/nipreps/nibabies), for various reasons (e.g., cost, effort, academic priorities), software development and dissemination often fall behind.

As the infant neuroimaging community matures, we expect that open science practices will become more fully embraced. Ultimately, openness between researchers, community building, and crosstalk between those with complementary skills will build needed standards (see *Q19*) and open science organically.

## 5 Limitations and conclusions

We presented brief responses to common questions from reviewers of infant neuroimaging work. While we focus primarily on the study of healthy, at-term infants, much of the information herein can be applied more broadly, for example to studies of toddlers or healthy premature infants. Special considerations exist for higher-risk groups (e.g., infants exposed to substances in-utero), which are not discussed here. Further, ethics or legal requirements may differ in non-U.S. and non-European countries, which are not reflected in the experiences of the authors and therefore not included. For more information on emerging work in developing countries see Turesky et al. (2019, 2020). Additionally, to maintain broad appeal and usefulness, we did not include the depth of detailed knowledge that exists in any one area, such as for fine-tuning specific MRI sequences. Indeed, a broad range of expertise is often required for designing and executing infant studies spanning developmental psychology, physics, statistics, engineering, and developmental neuroscience, and no single paper can explore all these facets in depth. Despite these limitations, we hope that these responses provide insight to the unique challenges associated with infant neuroimaging.

Like the very age group being studied, infant neuroimaging is a rapidly changing field and still developing its own unique set of standards. The field has come a long way in identifying special considerations for imaging infants. The common questions and collective responses shared here reflect that cumulative knowledge gathered from a number of developmental neuroimaging experts throughout the years. We hope that these responses aid researchers seeking to join the infant neuroimaging field as well as reviewers of infant study protocols, imaging grants, and manuscripts, fostering methodological transparency and bringing together a community to pave the way for the development of standards as the field matures.

## Declaration of Competing Interest

The authors declare that they have no known competing financial interests or personal relationships that could have appeared to influence the work reported in this paper.

## Data Availability

No data was used for the research described in the article.
