# Supplementary material for: Dear reviewers: Responses to common reviewer critiques about infant neuroimaging studies
Source: Dev Cogn Neurosci. 2021 Dec 27;53:101055. doi: 10.1016/j.dcn.2021.101055 (PMC8733260; doi:10.1016/j.dcn.2021.101055)
Supplement: Supplementary file 1 — Supplementary material [file mmc1.docx]

FIT’NG membership (in alphabetical order):

- **Zeena M. Ammar**
  Neuroscience Graduate Program
  Emory University
  Atlanta, GA, USA
- **Johanna Bick**Psychology Department
  University of Houston
  Houston, TX, USA
- **Rhodri Cusack**

Trinity College Institute of Neuroscience, Trinity College Dublin

- **Kelsey E. Davison**
  Boston University College of Health and Rehabilitation Sciences: Sargent College
  Boston, MA, USA
- **Jessica Dubois**
  Université de Paris, NeuroDiderot, Inserm, Paris, France
  Université Paris-Saclay, NeuroSpin-UNIACT, CEA, Gif-sur-Yvette, France
- **Aidan Ford**
  Neuroscience Graduate Program
  Emory University
  Atlanta, GA, USA
- **Nadine Gaab**
  Harvard Graduate School of Education, Cambridge, MA, USA
  Harvard Medical School, Boston, MA, USA
- **Simona Ghetti**Department of Psychology and Center for Mind and Brain
  University of California, Davis
  Davis, CA, USA
- **Kathryn L. Humphreys**
  Department of Psychology and Human Development
  Vanderbilt University
  Nashville, TN, USA
- **Jana Hutter**
  Biomedical Engineering Department
  King’s College London
  London, GB
- **Kathrine Skak Madsen**
  Danish Research Centre for Magnetic Resonance, Centre for Functional and Diagnostic Imaging and Research, Copenhagen University Hospital - Amager and Hvidovre
  Radiography, Department of Technology, University College Copenhagen
  Copenhagen, Denmark
- **Marjolein Mués**Department of Experimental, Clinical and Health Psychology
  Ghent University
  Ghent, Belgium
- **Cynthia Rogers**
  Departments of Psychiatry and Pediatrics
  Washington University School of Medicine
  St. Louis, MO, USA
- **Cristina Simon-Martinez**
  Institute of Information Systems, School of Management, HES-SO Valais-Wallis University of Applied Sciences and Arts Western Switzerland, Switzerland
- **Christopher D. Smyser**
  Departments of Neurology, Pediatrics, and Radiology
  Washington University in St. Louis
  St. Louis, MO, USA
- **Caroline Spencer**
  Department of Communication Sciences and Disorders
  Boston University
  Boston, MA, USA
- **Chad M. Sylvester**
  Department of Psychiatry
  Washington University in St. Louis
  St. Louis, MO, USA
- **Elina Thomas**
  Department of Psychiatry
  University of Vermont
  Burlington, VT, USA
- **Ted K. Turesky**
  Harvard Graduate School of Education
  Cambridge, MA, USA
- **Kelly A. Vaughn**
  University of Texas Health Science Center at Houston
  Houston, TX, USA
- **Sylia Wilson**
  Institute of Child Development
  University of Minnesota
  Minneapolis, MN, USA
- **Elizabeth Yen**
  Tufts University School of Medicine
  Mother Infant Research Institute at Tufts Medical Center
  Boston, MA, USA
- **Xi Yu**
  State Key Laboratory of Cognitive Neuroscience and Learning
  Beijing Normal University
  Beijing, China
- **Jennifer Zuk**
  Department of Speech, Language & Hearing Sciences
  Boston University
  Boston, MA, USA
